# Applying patient-reported outcome measures (PROMs) in physiotherapy: an evaluation based on the QUALITOUCH Activity Index

**DOI:** 10.1186/s40945-022-00152-3

**Published:** 2022-12-01

**Authors:** Mias Zaugg, Heiner Baur, Kai-Uwe Schmitt

**Affiliations:** 1 Dept. of Health Professions, Bern University of Applied Sciences (BFH), Academic-Practice-Partnership Between Insel Gruppe and BFH, Murtenstr. 10, 3008 Bern, Switzerland; 2 Dept. Health Sciences and Technology, Institute for Human Movement Science and Sport, ETH Zurich, Rämistr. 101, 8001 Zurich, Switzerland; 3 Dept. of Health Professions, Physiotherapy Research, Bern University of Applied Sciences (BFH), Murtenstr. 10, 3008 Bern, Switzerland

**Keywords:** Patient-Reported Outcome Measures, Physical Therapy, Quality Control, QUALITOUCH, Activity Index, Musculoskeletal Diseases, Biostatistics

## Abstract

**Background:**

Patient-reported outcome measures (PROMs) are tools to screen a population, to monitor the subjective progress of a therapy, to enable patient-centred care and to evaluate the quality of care. The QUALITOUCH Activity Index (AI) is such a tool, used in physiotherapy. This study aimed to provide reference values for expected AI outcomes.

**Methods:**

A large data set uniting clinical routine data and AI outcomes was generated; it consisted of data of 11,948 patients. For four defined diagnoses, i.e. chronic lower back pain, tibia posterior syndrome, knee joint osteoarthritis and shoulder impingement, the AI responses related to the dimensions “maximum pain level” and “household activity” were analyzed. Reference corridors for expected AI outcomes were derived as linear trend lines representing the mean, 1st and 3rd quartile.

**Results:**

Reference corridors for expected AI outcomes are provided. For chronic lower back pain, for example, the corridor indicates that the initial average AI value related to maximum pain of 49.3 ± 23.8 points on a visual analogue scale (VAS multiplied by factor 10) should be improved by a therapeutic intervention to 36.9 ± 23.8 points on a first follow-up after four weeks.

**Conclusions:**

For four exemplary diagnoses and two dimensions of the AI, one related to pain and one related to limitations in daily activities, reference corridors of expected therapeutic progress were established. These reference corridors can be used to compare an individual performance of a patient with the expected progress derived from a large data sample. Data-based monitoring of therapeutic success can assist in different aspects of planning and managing a therapy.

**Supplementary Information:**

The online version contains supplementary material available at 10.1186/s40945-022-00152-3.

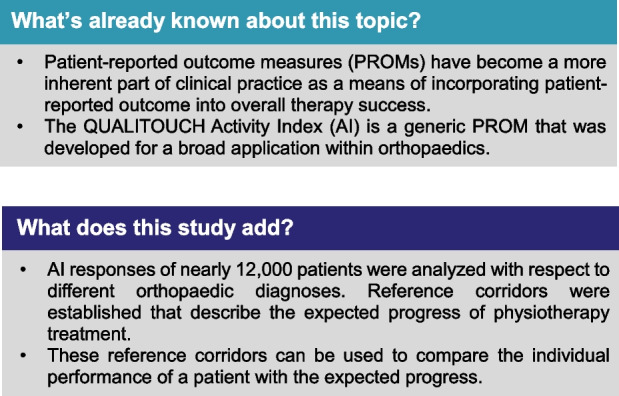



## Background

There are different ways to evaluate the success of a therapeutic intervention, to monitor the progress of a therapy and to gather expectations of patients, respectively. Those commonly used are clinical-based outcome measures, performance-based outcome measures, and patient-reported outcome measures (PROMs). In addition, there are patient-reported experience measures (PREMs) and patient-defined desired outcomes [1]. All of these measures can be regarded as indicators of different dimensions that can be used to evaluate the therapeutic outcome; Verburg et al., for example, presented a suggestion for using different dimensions to assess the outcome related to low back pain [2].

PROMs, in particular, have become a more inherent part of clinical practice as a means of incorporating patient-reported outcomes into overall therapy success [3]. PROMs are useful, for instance, for screening (e.g. to identify hidden topics), for monitoring (e.g. to evaluate intervention effectiveness and track a patient’s subjective outcome over time), for strengthening patient-centred care (e.g. to achieve better health outcomes and higher patient compliance) and for evaluating the quality of care (e.g. to assess strengths and weaknesses of certain therapies based on large data collections) [4].

Within the orthopaedics application there are condition-specific PROMs, such as WOMAC [5] and the Knee Injury and Osteoarthritis Outcome Score (KOOS) [6]. Other measures use more generic questionnaires to assess the health status or the quality of life, respectively: examples are the Short Form 36 (SF-36) [7] and the EuroQol Group 5-D Instrument (EQ-5D) [8]. The QUALITOUCH Activity Index (AI) [9] was designed as a generic, internet-based, patient-reported outcome measure to assure quality and to monitor therapy in orthopaedics and musculoskeletal diseases. It is provided by the QUALITOUCH HC Foundation (Zürich, Switzerland) [10] and consists of eight questions related to pain/symptoms, quality of sleep, limitation of daily activities, general health condition and therapy outcome (Table 1). The AI is used in different clinical settings related to musculoskeletal pathologies. Patients are provided with a link to the online questionnaire at the start of the therapy and then a follow-up is sent every four weeks until the end of the therapy. When completing the questionnaire for the first time the therapist supports the patient, whereas the follow-ups are carried out by the patient from home. The AI was originally developed in German. In contrast to other PROMs the AI does not yield a final overall score, but every single question must be evaluated on its own. Therefore, the AI captures physical limitations of the individual patient in different dimensions.

**Table 1:** Questions of the QUALITOUCH Activity Index. Translated from German to English by the authors for this publication (not validated)

Questions		Possible answers
Q1	How strong were your average pain levels or complaints over the last 24 h? (VAS 0–10)	no pain (0) to severe pain (10)
Q2	To what extent did pain or complaints affect your quality of sleep? (0/25/50/75/100%)	not at all/ slightly/ moderate/ strong/ extreme
Q3	How strongly did pain or complaints affect your household activities? (0/25/50/75/100%)	not at all/ slightly/ moderate/ strong/ extreme
Q4	How strongly did pain or complaints affect your leisure activities? (0/25/50/75/100%)	not at all/ slightly/ moderate/ strong/ extreme
Q5	How strongly did pain or complaints affect your work activities? (0/25/50/75/100%)	not at all/ slightly/ moderate/ strong/ extreme/ I do not work
Q6	Please rate your perceived general health condition (0/25/50/75/100%)	poor/ moderate/ good/ very good/ excellent
Q7	How satisfied are you with the therapy you have received? (100/66/33/0%)	fully/ moderate /little /not satisfied/ no information possible yet
Q8	How strong were your maximum pain levels or complaints over the past 24 h? (VAS 0–10)	no pain (0) to severe pain (10)

The AI was developed for a broad application in orthopaedics and musculoskeletal diseases, but it can also be applied for physiotherapy/physical therapy. Several studies report the application of the AI in the case of rheumatoid arthritis patients [11], to document the progress of lower back pain [9] and to measure the quality of treatment in interventional pain therapy [12].

However, if it is to have a wider practical use, then reference values, i.e. corridors of expected patient outcomes, would be helpful. The addition of reference values could enhance the efficacy of the AI as it would allow therapists to compare the performance of an individual patient with an expected “standard performance”.

The aim of this study was to investigate whether diagnosis-specific reference values for the AI could be derived statistically. The idea was to establish a reference that would allow an assessment as to whether the physiotherapy of an individual patient was progressing as expected, when compared to a large sample. The intention is to contribute to an evidence-based assessment of the therapeutic progress, which will in turn improve quality management in physiotherapy.

## Methods

The study aimed at developing statistically based reference values for the application of the AI. Four diagnoses at different body regions were chosen as clinical examples, all being of high clinical relevance, i.e. commonly seen in physiotherapy practice: chronic lower back pain (ICD-10: M54.4, M54.5, M54.8), tibia posterior syndrome (ICD-10: M77.5, M76.8, M21.4), knee joint arthrosis (ICD-10: M17.0, M17.1, M17.2, M17.4, M17.5, M17.9) and shoulder impingement (ICD-10: M75.4, M75.5).

### Study design

A retrospective study design using large samples of existing, fully anonymized clinical data was implemented. Data covering the period from 2010 to 2020 were available for analysis. While the AI data were collected by the QUALITOUCH HC Foundation, the clinical data were obtained from the two medical centres of the health-care provider Spiraldynamik® in Switzerland [13]. This health-care provider specializes in non-operative orthopaedic therapy of out-patients. Treatments related to the diagnoses chosen for this study generally involve patients receiving physiotherapy.

### Data sources

In a first step, the data received from the two sources were merged and pre-processed. Spiraldynamik® provided data referring to 21,183 individual patients. As some of the patients had visited the medical centre more than once within the 10-year period of our analysis, these 21,183 patients accounted for 54,131 medical cases. For each case there is a unique identifier in the clinical data set.

QUALITOUCH HC Foundation provided data for 12,106 patients who were treated at Spiraldynamik® and who were provided with the AI at least once. While some of these patients did not return the AI, many of them underwent therapy lasting more than one month, during which they were provided with the AI for follow-ups. This resulted in a total of 30,460 AI responses in this data set.

### Data sampling

**Fig. 1:**
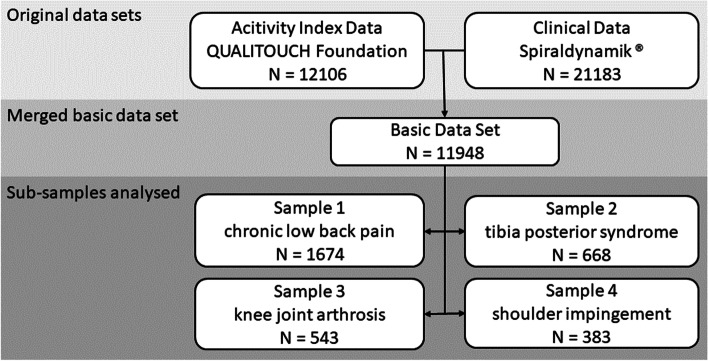
Data sets used in this study

The two data sets were merged into one basic data set, from which specific data sets were generated (Fig. 1). The patient ID served as a primary key in this process. Data were only included in the basic data set if the patient had returned at least one AI and had received at least one therapeutic consultation. The basic data set comprised a total of 11,948 patients. For each case of each patient the following parameters were included:

• Clinical data: age, sex, body mass index (BMI), diagnosis (coded according to ICD-10 German version);
• AI data: responses to questions Q1 – Q8 for the initial feedback and as many follow-ups as available.

From this basic data set, four sub-sets were derived, i.e. a separate data set was created for each of the chosen diagnoses.

### Data analysis

This paper focusses on question 1 (Q1) and question 4 (Q4) of the AI. These two questions are related to (maximum) pain (Q1) and to limitations to perform household activities (Q4). Note that in line with the definition of the AI in Q1 patients rate their (maximum) pain according to a visual analogue scale of 0 to 10; this value is then transferred to a point system by multiplying it with the factor 10. This results in all responses of the AI being of the same scale as, for example, Q4 is also given in values up to 100.

All data processing and all statistical analyses were conducted using the programme R-Studio (® 2009–2020 RStudio, PBC, Version 1.3.1093).

The AI responses were investigated with respect to their development over time. For each diagnosis the overall progress of the AI was analyzed. All available data at every point in time was used, i.e. the number of available responses differs from follow-up to follow-up. For statistical analysis it was defined that a minimum of 100 responses must be available at a point in time; follow-ups with less data were not considered. By comparing the responses of the last available follow-up to the initial AI response, the overall trend was determined for each case. By comparing all responses available at one follow-up to the initial response, the stepwise progress was analysed. To select the appropriate statistical test, QQ-plots were used to check for normal distribution. If the data were normally distributed, the t-test was used. If the data were not normally distributed, the Wilcoxon test was performed. For the statistical tests, a significance level of α = 0.05 was defined.

Finally, reference corridors were derived as a linear trend line using the results for the initial AI response and the follow-up responses. Like clinical percentile curves (e.g. [14–16]), the corridors are presented as scatterplots, with the mean, 1st and 3rd quartiles as linear models.

## Results

Table 2 summarizes the final data samples that were used in this study. A more detailed summary of the characteristics of the study population and the amount of data available at each follow-up is shown in the Additional file 1 (Table A1).

**Table 2:** Characteristics of the different samples

	**All diagnoses (basic data set)**	**Chronic lower back pain**	**Tibia posterior syndrome**	**Knee joint arthrosis**	**Shoulder impingement**
ICD-10	All	M54.4, M54.5, M54.8	M77.5, M76.8, M21.4	M17.0, M17.1, M17.2, M17.4, M17.5, M17.9	M75.4, M75.5
Patients [n]	11,948	1674	668	543	383
Cases [n]	37,356	7058	2229	1765	1455
Age [years] [average ± SD / min / max]	50.1 ± 16.2 /6.5 / 95.7	52.9 ± 15.0 /9.3 / 8.8	52.2 ± 14.8 /9.8 / 86.8	63.4 ± 9.9 /33.6 / 88.7	55.3 ± 11.9 /13.6 / 83.9
BMI [kg/m^2^] [average ± SD / min / max]	23.3 ± 3.8 /9.4 / 45.3	23.6 ± 3.8 /10.3 / 43.3	24.2 ± 4.3 /13.7 / 42.5	25.3 ± 4.3 /15.7 / 43.3	23.4 ± 3.4 /16.7 / 37.3
Sex (male/ female)	23% / 77%	23% / 77%	19% / 81%	22% / 78%	23% / 77%
Number of therapeutic consultations [n] [average ± SD, min. / max.]	4.1 ± 2.7 / 1, 19	4.4 ± 2.8 / 1, 13	4.4 ± 2.8 / 1, 14	4.4 ± 2.8 / 1, 10	4.5 ± 2.8 / 1, 14
Number of AI follow-ups [n] [average ± SD, min. / max.]	1.3 ± 1.9 / 0, 21	1.7 ± 2.1 / 0, 18	1.6 ± 2.1 / 0, 17	1.8 ± 2.3 / 0, 18	1.9 ± 2.4 / 0, 16

**Fig. 2:**
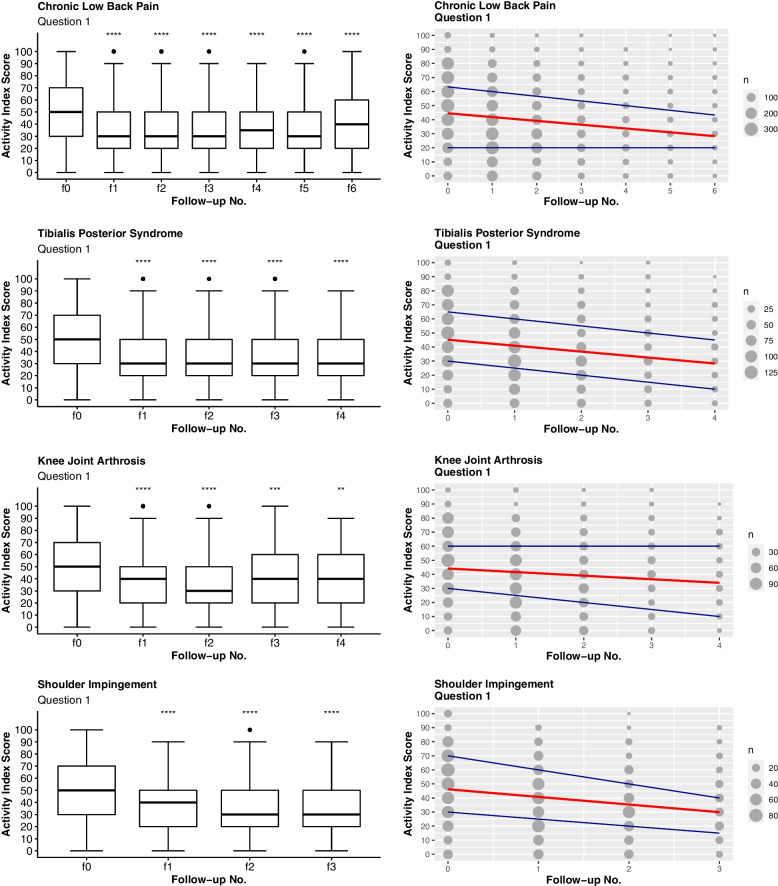
Results related to question 1 (Q1). On the left: boxplots with level of significance for the t-test comparing each follow-up number to f0 (ns: *p* > 0.05, *: *p* < 0.05, **: *p* < 0.01, ***: *p* < 0.001, ****: *p* < 0.0001). On the right: scatterplots with the mean, 1st and 3rd quartiles as linear models

QQ-plots showed a normal distribution for all samples. Thus, a t-test was used for further comparisons, i.e. for analyzing the AI responses at different follow-ups in relation to the baseline value at the start of the therapy. As can be seen in Fig. 2, for all diagnoses addressed here the AI indicated a significant improvement of the maximum pain levels (Q1) from baseline to follow-up. For chronic lower back pain, for example, the corridor indicates that an initial average AI value related to maximum pain of 49.3 ± 23.8 points should be expected to improve by a therapeutic intervention to 36.9 ± 23.8 points on a first follow-up after four weeks, and then further to 35.7 ± 23.0 points on a second follow-up after eight weeks, and so on. Similarly, an initial average AI value related to pain and limitations in household activities of 37.5 ± 24.7 points should be expected to improve to 27.6 ± 22.7 points on the first follow-up, and to 26.6 ± 22.0 points on a second follow-up.

**Fig. 3:**
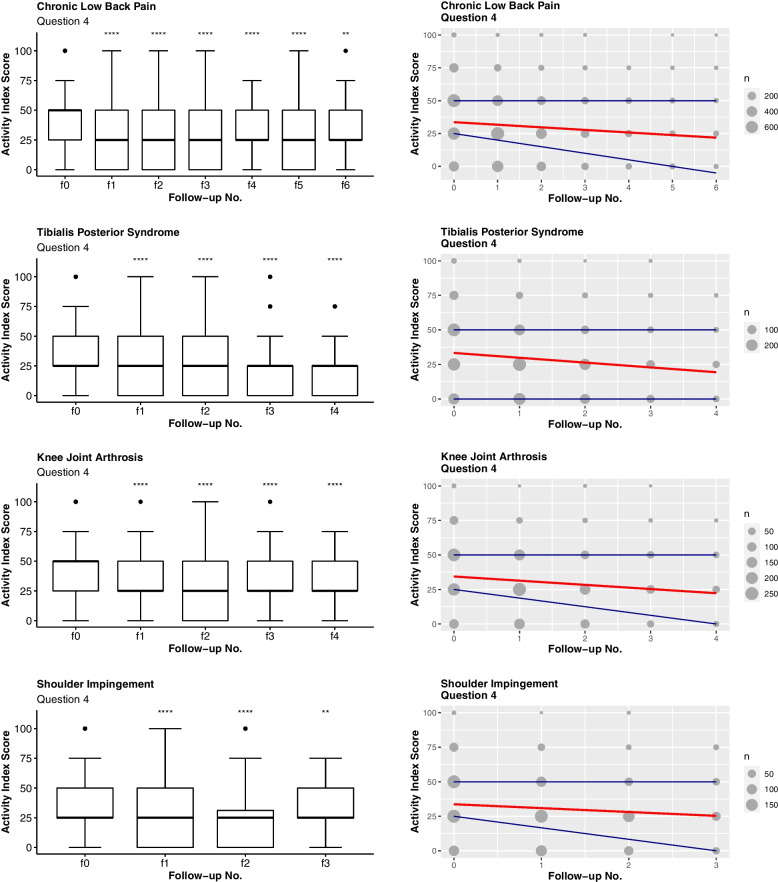
Results related to question 4 (Q4). On the left: boxplots with level of significance for the t-test comparing each follow-up number to f0 (ns: *p* > 0.05, *: *p* < 0.05, **: *p* < 0.01, ***: *p* < 0.001, ****: *p* < 0.0001). On the right: scatterplots with the mean, 1st and 3rd quartiles as linear models

Likewise, Fig. 3 documents a significant improvement in the ability to perform household activities reported by the patients (Q4). The therapeutic success is also shown in the corresponding scatterplots (Figs. 2 and 3, right). All scatterplots indicate a decreasing mean. The scatterplots also feature a corridor that is represented by the mean and the 1^st^ and 3^rd^ quartiles, i.e. an AI outcome within the corridor covers 50% of the responses.

## Discussion

In order to statistically derive diagnosis-specific reference values for the QUALITOUCH Activity Index (AI), two large data samples were successfully merged. The sample related to the AI was significantly smaller than the clinical data set, indicating that the AI was not issued and/or completed by all patients. Since the clinical partner also treats patients who are not the target population of the AI, this seems reasonable. Merging the data occasioned the loss of only a few entries, and these were all cases where the patient could not be identified in the clinical data set. Thus, the basic data set as derived here is as complete as possible and, due to its size, is regarded as a sound basis for further analysis, with high external validity and a strong informative value with regard to quality of care.

The patients included in the basic data set showed an average age of 50.1 years, an average BMI of 24.6 and a sex ratio of approximately one male to three females. The high proportion of women is remarkable, but age and BMI are in line with published data of the general Swiss population [17]. With respect to these overall descriptors, it can thus be assumed that the data reflect a representative population. The clearly increased age for the subsample of patients with knee osteoarthritis seems plausible given the degenerative nature of this pathology. However, additional characteristics that might have an influence on the therapeutic progress (e.g. education, occupation, comorbidities) could not be taken into account because those factors were not documented in either database.

For the statistical analysis, four different diagnoses were chosen. These cover different body regions, can be described as common fields of application in physiotherapy and require different therapeutic approaches. Therefore, it is believed that this choice can serve well as an example to demonstrate the impact of a generic PROM.

In contrast to other studies [18, 19], only one PROM was evaluated here, but it was evaluated with respect to several different diagnoses. The generic nature of the AI allowed this comparative analysis. Considering that physiotherapy is dealing with a variety of diagnoses in clinical practice, it seems reasonable and practical to use a single generic PROM instead of several diagnosis-specific ones. At the same time, this can be a limitation as analyses of diagnosis-specific aspects become more challenging, if not impossible. For this evaluation two dimensions of the AI were chosen, i.e. two questions. Both Q1 and Q4 are relevant for all patients. The maximum pain (Q1) was evaluated because it seems easier and more reliable to estimate than average pain (Q2). Discomfort during sleep (Q3) is known to be associated with pain and therefore was not used. Complaints during leisure time (Q5) and at work (Q6) were omitted in favour of focusing on household activities (Q4), which were deemed to represent some daily activity that is similarly relevant for patients of all age groups and socio-economic backgrounds.

As expected from other studies [9, 20], the AI did highlight a decline in pain and complaints after physiotherapy. For all diagnoses the AI documented a significant improvement between the first consultation and follow-up consultations. This confirms the assumption that the AI is a suitable instrument for recognising therapeutic progress and success.

The statistical procedure to derive reference corridors for expected AI progress over time was straightforward and rather simple, using a linear approximation. This reinforces the credibility and transparency of the results. The visualization as corridors allows for an easy comparison of the response of a specific patient with the statistical expectation. Hence, the corridors can be used as a monitoring tool to support both the therapist and the patient. In this way, it can be assessed whether the course of the therapy corresponds to the norm and whether it has an effect on the patient (per the different dimensions of the AI). This tool can thus quantify the effect of the therapy on the patient and complement the hands-on experience of the therapist.

Although the amount of data available was enormous, there were a few limitations in addition to those already mentioned above. In our data sample one patient can have multiple cases, and the AI questionnaire was issued for each different case, thus all were considered in the evaluation. This means that individual patients are represented several times, which could have an influence on the AI score (e.g. if chronically ill / with multiple diagnoses). Furthermore, many patients only filled in the AI at the beginning of their therapy, with a huge drop-off in the numbers of further follow-ups. To some extent this can be explained by the fact that some patients only needed a few sessions to complete their therapy and hence they stopped returning the AI at a follow-up. Others might have had poor compliance. From the data used here, it is unknown why any given patient stopped returning the AI. From a statistical point of view, while the declining number of responses from follow-up to follow-up can be explained, it does introduce uncertainties.

Besides these limitations, the established reference corridors offer a variety of opportunities related to quality of care. The use of PROMs involves the patient and contributes to considering the patient’s needs and identifying any unmet needs. Patients who are not responding well to therapy or whose success is stagnating can be identified early and options to adjust the therapy can be considered. This might also be helpful for decision-making, e.g. when weighing up conservative therapy versus surgical intervention. Using a reference corridor to compare the individual progress against a statistical expectation might help in this respect, and also in managing patient expectations. If, for example, a patient with knee joint arthrosis shows an AI score for Q1 of 70 points at the initial consultation, the reference corridor indicates that this patient is at the upper end of the statistical expectation. With this information the therapist can ensure the patient is closely monitored and if the score is reduced below 50 points at the third follow-up, the therapist is assured that such reduction represents the norm for this patient group indicating that the therapy seems to be successful whereas other patients only start a therapy with the same score. When using a PROM that covers different dimensions, as the generic AI does, a reference corridor can also be of help in prioritizing therapeutic aims and thus personalizing the intervention based on the expected outcome.

In addition to monitoring individual progress, quality control of an entire patient cohort from, for example, one physiotherapy practice is possible, and the therapeutic success of the practice can be documented and compared to the reference cohort. This enables practices to demonstrate the quality of their therapy, e.g. for health insurers [21], which is in line with current trends moving the health-care system towards a pay-for-performance system.

Future research should complement AI corridors for other diagnoses and provide corridors for further PROMs. Likewise, a predictive model in the form of a factor analysis could be a possibility to investigate in greater detail the predictive power of different influencing factors on such reference corridors. Table 2 already indicates several factors, such as age or BMI, that should be included in such a factor analysis. Further dimensions of the AI and medical aspects, such as comorbidity, should then also be included. Likewise, lifestyle related factors can be integrated to specify different peer groups to whom the reference corridors can be applied. The implementation of reference corridors in clinical practice and an evaluation of its impact can further contribute to discussion about evidence-based quality management in physiotherapy.

## Conclusions

Based on the evaluation of clinical data for a period of 11 years, this study demonstrated that PROMs have the potential to provide a basis for monitoring therapeutic progress. This evidence-based approach contributes to quality management in physiotherapy as it complements the hands-on experience of the therapist. A statistical approach using four exemplary diagnoses and two dimensions of the generic QUALITOUCH Activity Index – one related to pain and one related to limitations in daily activities daily activities – allowed us to establish reference corridors of expected progress. These reference corridors can be used to compare the individual performance of a patient to the expected progress based on a large sample of self-reported data. A data-based monitoring of the therapeutic success can assist in different aspects of planning and managing a therapy. It can, for instance, be consulted to manage patient expectations and to check for unmet needs, to identify and document more complex cases, to personalize the intervention to ensure it is centred on the patient’s needs, to address specific aspects in which an underperformance was recognized, or to help in deciding whether to continue a conservative path or consider surgery. Furthermore, such reference corridors can be useful for more general discussions in health care, for example, matters relating to compensation or the impact of novel therapeutic approaches.

## Supplementary information

**Additional file 1: Table A1.** Study population and number of data points considered in the evaluation. It was defined that a minimum of 100 responses was required to include the follow-up in the statistical analysis.

## Data Availability

The data sets generated and/or analyzed during the current study are not publicly available but can be made available from the corresponding author on reasonable request and if agreed by the clinical partners.

## Abbreviations

AI: QUALITOUCH Activity Index, the AI is a questionnaire consisting of eight items
BMI: Body mass index
PREM: Patient-reported experience measures
PROM: Patient-reported outcome measures
