# Supplementary material for: Applying patient-reported outcome measures (PROMs) in physiotherapy: an evaluation based on the QUALITOUCH Activity Index
Source: Arch Physiother. 2022 Dec 1;12:27. doi: 10.1186/s40945-022-00152-3 (PMC9713991; doi:10.1186/s40945-022-00152-3)
Supplement: Supplementary file 1 — Additional file 1: Table A1. Study population and number of data points considered in the evaluation. It was defined that a minimum of 100 responses was required to include the follow-up in the statistical analysis. [file 40945_2022_152_MOESM1_ESM.pdf]

Appendix

Table A1: Study population and number of data points considered in the evaluation. It was defined that a minimum of 100 responses was required to include the follow-up in the statistical analysis.

	Follow-up					
	T1	T2	T3	T4	T5	T6
All diagnoses (basic data set)						
Age [average ± SD / min / max]	52.62 ± 15.30 / 8.17 / 93.85	54.06 ± 14.77 / 9.27 / 93.85	55.54 ± 14.35 / 11.39 / 93.85	56.55 ± 14.07 / 13.62 / 90.91	57.35 ± 13.91 / 13.62 / 90.91	57.78 ± 13.94 / 13.62 / 90.91
BMI [average ± SD / min / max]	23.31 ± 3.79 / 9.4 / 45.30	23.41 ± 3.83 / 9.40 / 45.20	23.46 ± 3.78 / 10.30 / 42.00	23.51 ± 3.68 / 15.70 / 42.00	23.45 ± 3.63 / 15.70 / 38.70	23.28 ± 3.64 / 15.70 / 38.70
Sex (male / female)	20% / 80%	19% / 81%	18% / 82%	18% / 82%	17% / 83%	19% / 81%
AI, Question 1 [n]	8331	4236	1823	978	524	322
p value (t-test) for comparison of the AI score relative to f0	0.00	0.00	0.00	0.00	0.00	0.00
AI, Question 4 [n]	8269	4196	1804	966	513	319
p value (t-test) for comparison of the AI score relative to f0	0.00	0.00	0.00	0.00	0.00	0.00
Chronic lower back pain						
Age [average ± SD / min / max]	52.87 ± 14.98 / 9.27 / 88.19	54.09 ± 14.54 / 9.27 / 88.19	55.38 ± 14.12 / 14.25 / 86.90	55.89 ± 13.74 / 14.86 / 86.90	56.39 ± 13.72 / 14.86 / 86.90	55.49 ± 14.46 / 14.86 / 86.90
BMI [average ± SD / min / max]	23.59 ± 3.76 / 10.30 / 43.30	23.67 ± 3.74 / 10.30 / 43.30	23.75 ± 3.73 / 10.30 / 38.70	23.85 ± 3.71 / 17.40 / 38.70	23.88 ± 3.76 / 17.40 / 38.70	23.53 ± 3.71 / 17.80 / 38.70
Sex (male / female)	24% / 76%	29% / 71%	20% / 80%	19% / 81%	18% / 82%	21% / 79%
AI, Question 1 [n]	1822	1041	524	302	188	117
p value (t-test) for comparison of the AI score relative to f0	0.00	0.00	0.00	0.00	0.00	0.00
AI, Question 4 [n]	1803	1027	519	300	184	115
p value (t-test) for comparison of the AI score relative to f0	0.00	0.00	0.00	0.00	0.00	0.00
Tibia posterior syndrome						
Age [average ± SD / min / max]	52.19 ± 14.75 / 9.80 / 86.82	53.49 ± 13.92 / 13.17 / 84.01	53.70 ± 14.50 / 14.64 / 79.90	53.95 ± 14.89 / 14.64 / 79.90		
BMI [average ± SD / min / max]	24.22 ± 4.28 / 13.70 / 42.50	24.64 ± 4.66 / 16.40 / 42.50	24.07 ± 4.24 / 16.40 / 37.60	24.47 ± 4.40 / 17.30 / 37.30		
Sex (male / female)	19% / 81%	16% / 84%	17% / 83%	18% / 82%		
AI, Question 1 [n]	743	441	213	114		
p value (t-test) for comparison of the AI score relative to f0	0.00	0.00	0.00	0.00		
AI, Question 4 [n]	738	433	213	112		
p value (t-test) for comparison of the AI score relative to f0	0.00	0.00	0.00	0.00		
Knee joint arthrosis						
Age [average ± SD / min / max]	63.41 ± 9.87 / 33.56 / 88.72	64.07 ± 9.30 / 33.56 / 87.35	64.84 ± 9.65 / 40.55 / 85.73	64.32 ± 9.47 / 41.01 / 84.93		
BMI [average ± SD / min / max]	25.31 ± 4.32 / 15.70 / 43.30	25.26 ± 4.45 / 15.70 / 43.30	25.27 ± 4.31 / 17.70 / 42.00	25.53 ± 4.65 / 17.70 / 42.00		
Sex (male / female)	23% / 77%	19% / 81%	18% / 82%	21% / 79%		
AI, Question 1 [n]	596	335	174	103		
p value (t-test) for comparison of the AI score relative to f0	0.00	0.00	0.00	0.00		

AI, Question 4 [n]	591	331	174	102		
p value (t-test) for comparison of the AI score relative to f0	0.00	0.00	0.00	0.00		
Shoulder impingement						
Age [average ± SD / min / max]	55.34 ± 11.91 / 13.62 / 83.87	55.48 ± 11.93 / 13.62 / 81.25	55.85 ± 10.83 / 13.62 / 81.25			
BMI [average ± SD / min / max]	23.36 ± 3.41 / 16.70 / 37.30	23.29 ± 3.33 / 16.70 / 37.30	23.16 ± 3.23 / 17.50 / 33.40			
Sex (male / female)	24% / 76%	25% / 75%	24% / 76%			
AI, Question 1 [n]	454	295	158			
p value (t-test) for comparison of the AI score relative to f0	0.00	0.00	0.00			
AI, Question 4 [n]	451	292	156			
p value (t-test) for comparison of the AI score relative to f0	0.00	0.00	0.01			
